# Clinical outcomes of the implementation of acute care surgery system in South Korea: a multi‐centre, retrospective cohort study

**DOI:** 10.1111/ans.19366

**Published:** 2024-12-17

**Authors:** Gun‐Hee Yi, Suk‐Kyung Hong, Yang‐Hee Jun, Sungyeon Yoo, Jung‐Min Bae, Keesang Yoo, Yun Tae Jung, EunYoung Kim, Narae Lee, Min Jung Ko, Hogyun Shin, Hak‐Jae Lee

**Affiliations:** ^1^ Division of Acute Care Surgery, Department of Surgery University of Ulsan College of Medicine, Asan Medical Center Seoul Korea; ^2^ Department of Surgery Yeungnam University College of Medicine Daegu Korea; ^3^ Division of Acute Care Surgery, Department of Surgery, Samsung Medical Center Sungkyunkwan University School of Medicine Seoul Korea; ^4^ Department of Surgery, Gangneung Asan Hospital University of Ulsan College of Medicine Gangneung Korea; ^5^ Division of Trauma and Surgical Critical Care, Department of Surgery, Seoul St. Mary's Hospital, College of Medicine The Catholic University Seoul Korea; ^6^ Division of Healthcare Technology Assessment Research National Evidence‐based Healthcare Collaborating Agency Seoul Korea

**Keywords:** abdomen, acute care surgery, operating rooms, postoperative complications, tertiary care centres

## Abstract

**Background:**

In emergency general surgery (EGS), rapid judgement and prompt emergency surgery play a significant role in determining the patient's prognosis. This study aimed to evaluate whether implementing the acute care surgery (ACS) system in Korea has improved the clinical outcomes of patients.

**Methods:**

This retrospective cohort study was conducted at three tertiary hospitals in Korea. The study included patients aged 18–99 years who required EGS due to acute abdomen or abdominal trauma. A window period of 4 months was set after the implementation of the ACS system, and the clinical outcomes before and after the implementation were compared.

**Results:**

A total of 2146 patients were enrolled in the study, with 1008 in the pre‐ACS group and 1138 in the post‐ACS group. After propensity score matching, 901 patients were selected in the pre‐ACS group and 906 patients were selected in the post‐ACS group. The time from emergency room admission to operating room transfer was reduced in the post‐ACS group, with a mean of 452.2 ± 347.0 min compared to 522.1 ± 416.5 min in the pre ACS group (*P* = 0.001). Moreover, the complication rates were reduced in the post‐ACS group (38.3% vs. 31.3%, *P* = 0.006).

**Conclusions:**

The implementation of the ACS system can lead to faster surgical decision‐making and the prompt execution of emergency surgery for patients, thereby reducing postoperative complications.

## Graphical abstract

This study evaluates the impact of implementing the acute care surgery (ACS) system in South Korea. Conducted across three tertiary hospitals, the results show reduced time from emergency room admission to surgery and lower postoperative complication rates. The findings suggest that the ACS system improves surgical decision‐making and patient outcomes.

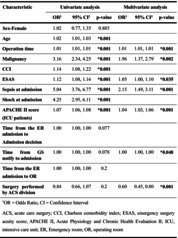

## Introduction

Advances in general emergency surgery have led to the development of various tools and implementation practices to ensure the appropriate treatment of patients in emergency care. Introducing systems such as the emergency medicine specialists certification system and establishing trauma centres can help improve patient outcomes.^1^ Moreover, regarding emergency patient care, time is one of the most crucial factors. Numerous studies have indicated that the initial hours following either trauma or the onset of acute illness are critical for determining patient outcomes.^2^, ^3^ Accordingly, surges in the demand for timely and appropriate medical services have exacerbated existing challenges due to a persistent absence of medical personnel and systems.

Recognizing this challenge, representatives from various American trauma‐related associations, such as the American College of Surgeons, the Association for the Surgery of Trauma, the Eastern Association for the Surgery of Trauma, and the Western Trauma Association, convened in 2003 to discuss training procedures and tasks regarding the acute care surgery (ACS) system. Before the ACS system was implemented, doctors performed emergency surgeries and emergency room (ER) consultations according to a predetermined schedule. Furthermore, these duties were managed in addition to their elective surgeries and outpatient appointments, leading to a heavy workload, often resulting in delays in emergency surgeries. In 2005, the ACS model was proposed, which has since been adopted in most countries.^4^, ^5^, ^6^, ^7^, ^8^ The ACS system, in which doctors specifically assigned to emergency surgery are on standby to provide immediate and appropriate treatment, including intensive care, was developed to reduce the workload of the traditional on‐call system (TROS), which similarly handles elective surgeries, outpatient treatments, and emergency surgeries. The need for the ACS system arose due to a lack of residents and an increasing demand for improved quality of emergency surgical care. A system where specialists are on standby in the ER can potentially shorten the time required to conduct suitable diagnostic tests and determine a treatment plan. Moreover, the fact that specialized doctors can perform surgeries directly can minimize time wasted. In addition to these benefits, the increasing sophistication and specialization in the treatment of trauma and abdominal organ injuries have also eased the workload for surgeons who are not specialized in this field.

Every year in the United States, over 3 million patients are hospitalized due to emergency general surgery (EGS) conditions.^9^ For EGS cases, particularly those necessitating surgical intervention, deciding on a treatment strategy promptly and carrying out surgical treatment rapidly are critical in determining the patient's prognosis. According to the comparative studies conducted before and after implementing the ACS system for representative surgical diseases like acute appendicitis and acute cholecystitis, there has been a statistically significant reduction in the time to operating room (OR) transfer.^10^, ^11^ Studies have found that the assertive and rapid implementation of treatments can reduce postoperative complications and shorten hospital stays, ultimately reducing medical costs.^4^, ^6^, ^12^, ^13^, ^14^ In Korea, a country with a critical issue of people avoiding choosing surgery as a profession, which has led to staff shortages and the overworking of existing staff, the ACS system has been gradually introduced since the mid‐2010s.^10^ However, while numerous studies have been conducted to validate the usefulness of the ACS system from single‐centre studies, evidence from multi‐centre studies are lacking.^4^, ^6^, ^15^ Therefore, the present study investigated the clinical utility, stability, and effectiveness of the ACS system in three tertiary hospitals where the implementation of the ACS system in Korea was well‐defined and systematically managed.

## Methods

### Patients selection and ACS team structure

This was a retrospective cohort study conducted at three tertiary hospitals, including Asan Medical Center (AMC), Samsung Medical Center (SMC), and Yeungnam University College of Medicine Hospital (YUCMH). The characteristics of the three tertiary hospitals participating in the study are presented in Table 1. This study included patients aged 18–99 years who required EGS due to acute abdomen or abdominal trauma, including conditions such as acute appendicitis, cholecystitis, bowel obstruction, and bowel infarction, perianal abscess, Fournier's gangrene, wound evisceration, and spleen bleeding. The exclusion criteria were: (i) patients under 18 years of age; (ii) patients who underwent emergency surgery from departments other than the surgery department; (iii) patients admitted to the ward and initially treated conservatively before surgery; and (iii) cases involving vascular surgery or organ transplantation. Additionally, surgeries conducted in the first 4 months immediately after the ACS system implementation at each hospital were excluded. For AMC, the system was introduced in March 2017. Patients from the pre‐ACS period (from 1 July 2014 to 31 December 2016) and the post‐ACS period (from 1 July 2017 to 31 December 2019) were included in this study. For both SMC and YUCMH, the system was introduced in March 2018. Hence, our study considered patients from the pre‐ACS period of 1 July 2016, to 31 December 2017, and the post‐ACS period of 1 July 2018, to 31 December 2019. In this study, we set a window period for the ACS system to be well‐established and focused solely on surgeries conducted after that period. The patients' general characteristics and medical histories were accessed from the electronic medical record. Data access for research purposes was conducted in March 2023, and the authors were unable to identify individual participants after data collection.

**Table 1:** Acute care surgery (ACS) system of three hospitals participating in the study

Hospital	Number of beds in the hospital	Number of elective surgeries per day	Number of ACS doctors dedicated to emergency surgery	Operating room dedicated to emergency surgery	Working types of ACS staff
AMC	2736	80–90	5	No	In‐hospital system
SMC	2168	45–55	4	No	In‐hospital system
YUCMH	1002	10–20	2	No	On‐call system

The ACS team was composed of surgeons who completed residency training and specialized in ACS, lower gastrointestinal surgery, upper gastrointestinal surgery, or hepatobiliary surgery. Initial patient assessments were typically performed by residents, while board‐certified surgeons primarily handled subsequent patient management, including decision‐making and performance of surgeries. This structure provided continuous coverage, ensuring care was available 24 h a day, 365 days a year.

The Institutional Review Board of the National Evidence‐Based Healthcare Collaborating Agency approved this study (NECA‐IRB no. NECAIRB22‐004) and waived the requirement for informed consent.

### Outcomes

The primary outcome was the time from ER presentation to arrival at the OR. The secondary outcomes included: (i) the time from ER presentation to the decision of admission by the surgeon; (ii) the length of hospital and intensive care unit (ICU) stay; (iii) discharge statuses, such as regular discharge, death, or transfers; (iv) re‐admission and death within 30 days after surgery; (v) need for re‐operation; (vi) complication rate; and (vii) mortality rate. The emergency surgery acuity score (ESAS) was utilized to predict the mortality associated with emergency surgery.^16^ Additionally, postoperative complications were classified using the Clavien–Dindo classification system.^17^

### Statistical analysis

The chi‐square or Fisher's exact test was performed for categorical variables, while the Student's *t*‐test or Mann–Whitney *U* test was performed for continuous variables to compare the baseline characteristics before and after the introduction of the ACS system, the chi‐square or Fisher's exact test was performed for categorical variables. To reduce the effects of selection bias and potential confounding variables, we employed propensity score matching. In this analysis, considering variations among different medical institutions, we treated them as random effects. The propensity scores included characteristics of the subjects: sex, age, type of surgery, total operation time, the specialty conducting the surgery, whether it was a regular surgery, surgical site, cause of surgery, type of surgery, diagnosis, presence of cancer, Charlson comorbidity index (CCI), ESAS, and shock status. The scores were derived using logistic regression, and matching was performed according to the ‘nearest neighbor’ method using a 0.1‐width calliper and at a 1:1 ratio. The level of statistical significance was set at *P* < 0.05. Univariate analyses were conducted by calculating odds ratios to identify statistically significant candidate factors relating to mobility decline. Variables with *P* < 0.2 were selected for use in the multivariate analyses. Significant independent variables were identified using a stepwise selection process based on the Akaike Information Criterion (AIC) in a multivariate backward logistic regression analysis to predict Clavien–Dindo classification 3 or higher complications. This process ensured that only variables contributing significantly to the model according to AIC were included without explicitly applying a significance level threshold for inclusion in the final model. Statistical analyses were conducted using R software (version 4.0.3; R Foundation for Statistical Computing, Vienna, Austria; http://www.r-project.org).

## Results

Overall, 2146 patients were enrolled in the study, with 1008 and 1138 in the pre‐ACS and post‐ACS groups, respectively. Upon examining the baseline characteristics, age disparities, cause of diagnosis, Acute Physiology and Chronic Health Evaluation II (APACHE II) score, ICU admission rates, affected major organs, cause of emergency surgery, and the surgical departments involved showed statistical differences.

The mean age of the pre‐ACS group was 58.2 ± 17.4 years, whereas the post‐ACS group had a slightly older mean age of 60.4 ± 17.6 years (*P* = 0.005). The number of cases diagnosed as non‐malignant and malignant in the pre‐ACS group was 798 (79.2%) and 210 (20.8%), respectively. In contrast, the cases diagnosed as non‐malignant and malignant in the post‐ACS group were 956 (84.0%) and 182 (16.0%), respectively, showing a significantly higher proportion of non‐malignancy diagnoses in the post‐ACS group (*P* = 0.006).

In the ICU‐admitted patients, the pre‐ACS group had an APACHE II score of 17.1 ± 8.3 and an admission rate of 29.1% (293 patients), while the post‐ACS group had an APACHE II score of 19.5 ± 8.8 with a 37.0% admission rate (421 patients). Both differences were statistically significant (both *P* = 0.001). Additionally, after the introduction of the ACS system, surgeries were primarily conducted in the ACS division (Table 2).

**Table 2:** Baseline characteristics of the patients (*n* = 2146)

Characteristics	Pre ACS group (*n* = 1008)	Post ACS group (*n* = 1138)	*P*‐value
Age (years)	58.2 ± 17.4	60.4 ± 17.6	* **0.005**
Men (*n*, %)	583 (57.8%)	660 (58.0%)	0.975
Cause of diagnosis (*n*, %)			* **0.006**
Non‐malignancy	798 (79.2%)	956 (84.0%)	
Malignancy	210 (20.8%)	182 (16.0%)	
Operation type (*n*, %)			0.238
Open surgery	621 (61.6%)	661 (58.1%)	
Laparoscopic surgery	371 (36.8%)	455 (40.0%)	
Others	16 (1.6%)	22 (1.9%)	
Charlson Comorbidity Index	1.2 ± 2.0	1.1 ± 1.9	0.239
ESAS	2.9 ± 3.4	2.9 ± 3.4	0.824
APACHE II score (in ICU‐admitted patients)	17.1 ± 8.3	19.5 ± 8.8	* **0.001**
Sepsis at admission (*n*, %)	167 (16.6%)	204 (17.9%)	0.439
Shock at admission (*n*, %)	85 (8.4%)	89 (7.8%)	0.661
ICU admission (*n*, %)	293 (29.1%)	421 (37.0%)	* **0.001**
Involved major organ (*n*, %)			* **0.001**
Stomach	58 (5.8%)	83 (7.3%)	
Small intestine	328 (32.5%)	361 (31.7%)	
Large intestine	169 (16.8%)	261 (22.9%)	
Gall bladder	109 (10.8%)	52 (4.6%)	
Appendix	288 (28.6%)	305 (26.8%)	
Others	56 (5.6%)	76 (6.7%)	
Causes of emergency surgery (*n*, %)			* **0.001**
Obstruction	154 (15.3%)	160 (14.1%)	
Perforation	304 (30.2%)	410 (36.0%)	
Ischemia/infarct	75 (7.4%)	84 (7.4%)	
Hernia	38 (3.8%)	35 (3.1%)	
Acute appendicitis	285 (28.3%)	304 (26.7%)	
Acute cholecystitis	109 (10.8%)	52 (4.6%)	
Others	43 (4.3%)	93 (8.2%)	
Divisions performing surgery (*n*, %)			* **0.001**
Acute care surgery (ACS)	58 (5.8%)	756 (66.4%)	
Upper GI (ST)	174 (17.3%)	81 (7.1%)	
Lower GI (CRS)	421 (41.8%)	202 (17.8%)	
Hepatobiliary (HBP)	265 (26.3%)	83 (7.3%)	
Others (Breast, Endocrine, *etc*.)	90 (8.9%)	16 (1.4%)	

*Note*: Values are expressed as mean ± standard deviation or number and percentage (*n*, %).

Abbreviations: ACS, acute care surgery; APACHE II, Acute Physiology and Chronic Health Evaluation II; CRS, colorectal surgery; ESAS, emergency surgery acuity score; GI, gastrointestinal; HBP, hepatobiliary surgery; ICU, intensive care unit; ST, stomach surgery.

* Indicates statistical significance, *P* < 0.05.

Regarding the analysis of the propensity score matching, some areas showed differences from before the matching (Table 3). After propensity matching, 901 and 906 patients were selected for the pre‐ACS and post‐ACS groups, respectively. No statistically significant difference other than the APACHE II score and the ICU admission rate was observed.

**Table 3:** Baseline characteristics of the patients after propensity score matching (*n* = 1807)

Characteristics	Pre ACS group (*n* = 901)	Post ACS group (*n* = 906)	*P*‐value
Age (years)	58.4 ± 17.6	58.8 ± 17.6	0.617
Men (*n*, %)	529 (58.7%)	528 (58.3%)	0.889
Cause of diagnosis (*n*, %)			0.812
Non‐malignancy	727 (80.7%)	735 (81.1%)	
Malignancy	174 (19.3%)	171 (18.9%)	
Operation type (*n*, %)			0.708
Open surgery	547 (60.7%)	558 (61.6%)	
Laparoscopic surgery	338 (37.5%)	328 (36.2%)	
Others	16 (1.8%)	20 (2.2%)	
Charlson Comorbidity Index	1.2 ± 2.0	1.2 ± 1.9	0.745
ESAS	2.9 ± 3.4	2.9 ± 3.4	0.823
APACHE II score (in ICU‐admitted patients)	17.2 ± 8.3	19.1 ± 8.9	* **0.008**
Sepsis at admission (*n*, %)	150 (16.6%)	158 (17.4%)	0.701
Shock at admission (*n*, %)	76 (8.4%)	77 (8.5%)	1.000
ICU admission (*n*, %)	267 (29.6%)	321 (35.4%)	* **0.010**
Involved major organ (*n*, %)			0.842
Stomach	56 (6.2%)	57 (6.3%)	
Small intestine	299 (33.2%)	292 (32.2%)	
Large intestine	165 (18.3%)	175 (19.3%)	
Gall bladder	66 (7.3%)	52 (5.7%)	
Appendix	262 (29.1%)	276 (30.5%)	
Others	53 (5.8%)	54 (5.7%)	
Causes of emergency surgery (*n*, %)			0.807
Obstruction	138 (15.3%)	132 (14.6%)	
Perforation	293 (32.5%)	299 (33.0%)	
Ischemia/infarct	70 (7.8%)	75 (8.3%)	
Hernia	34 (3.8%)	31 (3.4%)	
Acute appendicitis	259 (28.7%)	277 (30.6%)	
Acute cholecystitis	66 (7.3%)	52 (5.7%)	
Others	41 (4.5%)	40 (4.4%)	
Divisions performing surgery (*n*, %)			* **0.001**
Acute care surgery (ACS)	54 (6.0%)	605 (66.8%)	
Upper GI (ST)	163 (18.1%)	59 (6.5%)	
Lower GI (CRS)	398 (44.2%)	154 (17.0%)	
Hepatobiliary (HBP)	205 (22.8%)	76 (8.4%)	
Others (Breast, Endocrine, *etc*.)	81 (9.0%)	12 (1.3%)	

*Note*: Values are expressed as mean ± standard deviation or number and percentage (*n*, %).

Abbreviations: ACS, acute care surgery; APACHE II, Acute Physiology and Chronic Health Evaluation II; CRS, colorectal surgery; ESAS, emergency surgery acuity score; GI, gastrointestinal; HBP, hepatobiliary surgery; ICU, intensive care unit; ST, stomach surgery.

* Indicates statistical significance, *P* < 0.05.

The clinical outcomes before and after the introduction of the ACS system are summarized in Table 4. The data reveals that the time from ER admission to transfer to the OR was reduced in the post‐ACS group, with a mean of 452.2 ± 347.0 min compared with 522.1 ± 416.5 min in the pre‐ACS group (*P* = 0.001); the operation time (Optime) in the post‐ACS group had a mean of 132.7 ± 65.9 min in contrast to the pre‐ACS group's mean of 139.2 ± 65.6 min (*P* = 0.034). Moreover, notable shifts in surgery timings between the groups were observed. The pre‐ACS group had more surgeries during weekday nights (45.0%), while the post‐ACS group exhibited a rise in weekend surgeries (37.1%), with a significant difference (*P* = 0.001).

**Table 4:** Pre‐ and post‐ACS group clinical outcomes after propensity score matching (*n* = 1807)

Characteristics	Pre ACS group (*n* = 901)	Post ACS group (*n* = 906)	*P*‐value
Primary outcome			
Time from the ER admission to the OR (min)	522.1 ± 416.5	452.2 ± 347.0	* **0.001**
Secondary outcomes			
Operation time (min)	139.2 ± 65.6	132.7 ± 65.9	* **0.034**
Period that the surgery was performed (*n*, %)			* **0.001**
Weekday daytime (7 am–6 pm)	272 (30.2%)	246 (27.2%)	
Weekday night‐time (6 pm–7 am)	405 (45.0%)	324 (35.8%)	
Weekend (Sat–Sun)	224 (24.9%)	336 (37.1%)	
Length of hospital stay (days)	14.4 ± 16.6	14.0 ± 18.5	0.574
Length of ICU stay (days)	7.3 ± 13.3	7.9 ± 14.2	0.583
Time from the ER admission to decision of admission (min)	377.6 ± 361.7	324.0 ± 340.5	* **0.001**
Re‐operation during hospital stay (*n*, %)	56 (6.2%)	66 (7.3%)	0.417
Re‐admission (within 30 days after hospital discharge) (*n*, %)	42 (4.7%)	43 (4.7%)	1.000
Clavien–Dindo classification (*n*, %)			* **0.006**
No complication (0)	556 (61.7%)	623 (68.8%)	
Mild complication (1–2)	238 (26.4%)	190 (21.0%)	
Severe complication (3–5)	107 (11.9%)	93 (10.3%)	
In‐hospital mortality (*n*, %)	26 (2.9%)	39 (4.3%)	0.135

*Note*: Values are expressed as mean ± standard deviation or number and percentage (*n*, %).

Abbreviations: ER, emergency room; ICU, intensive care unit;OR, operating room.

* Indicates statistical significance, *P* < 0.05.

Upon analysing complications based on the Clavien–Dindo classification, we found a significant difference in the overall rates of complications between the groups. Specifically, a higher proportion of patients in the pre‐ACS group (38.3%) experienced complications compared with those in the post‐ACS group (31.3%), with a *P*‐value of 0.006. This suggests an overall decrease in complications following the ACS implementation. Other outcomes, such as the length of hospital stay, length of ICU stay, time from ER admission to the decision of admission by the surgeon, re‐operation during hospital stay, re‐admission rates, and in‐hospital mortality, did not show statistically significant differences between the two groups.

Univariate analysis revealed statistically significant associations between the occurrence of Clavien–Dindo classification 3 or higher complications and various factors, including age, operation time, the presence of malignancy, CCI, ESAS, sepsis at admission, shock at admission, APACHE II score, time from ER admission to admission decision, time from general surgery notification to admission, and time from ER admission to OR transfer.

Furthermore, multivariate logistic regression analysis identified operation time, the presence of malignancy, ESAS, sepsis at admission, APACHE II score, time from general surgery notification to admission, and surgeries conducted by the ACS division as statistically significant predictors (Table 5).

**Table 5:** Univariate and multivariate logistic regression analyses to identify prognostic factors influencing the occurrence of Clavien–Dindo Grade 3 or higher complications

Characteristic	Univariate analysis			Multivariate analysis		
	OR^a^	95% CI^a^	*P*‐value	OR^a^	95% CI^a^	*P*‐value
Sex: Female	1.02	0.77, 1.35	0.885			
Age	1.02	1.01, 1.03	* **0.001**			
Operation time	1.01	1.01, 1.01	* **0.001**	1.01	1.01, 1.01	* **0.001**
Malignancy	3.16	2.34, 4.25	* **0.001**	1.96	1.37, 2.79	* **0.002**
CCI	1.14	1.08, 1.22	* **0.001**			
ESAS	1.12	1.08, 1.16	* **0.001**	1.05	1.00, 1.10	* **0.035**
Sepsis at admission	5.04	3.76, 6.77	* **0.001**	2.15	1.49, 3.11	* **0.001**
Shock at admission	4.25	2.95, 6.11	* **0.001**			
APACHE II score (ICU patients)	1.07	1.06, 1.08	* **0.001**	1.04	1.03, 1.06	* **0.001**
Time from the ER admission to admission decision	1.00	1.00, 1.00	0.077			
Time from GS notify to admission	1.00	1.00, 1.00	0.078	1.00	1.00, 1.00	* **0.048**
Time from the ER admission to OR	1.00	1.00, 1.00	0.2			
Surgery performed by ACS division	0.84	0.66, 1.07	0.2	0.60	0.45, 0.80	* **0.001**

^a^ CI, confidence interval; OR, odds ratio.

Abbreviations: ACS, acute care surgery; APACHE II, Acute Physiology and Chronic Health Evaluation II; CCI, Charlson comorbidity index; ER, Emergency room; ESAS, emergency surgery acuity score; ICU, intensive care unit; OR, operating room.

* Indicates statistical significance, *P* < 0.05.

## Discussion

The timing of surgical intervention is crucial for patients requiring EGS. In most cases, prompt surgical intervention is considered beneficial for the patient's outcomes. However, the urgency of surgical conditions requiring emergency surgery varies in their acuity. Some conditions need immediate intervention, while others can be managed within 1–2 days. Ultimately, surgical treatment is the primary therapy, and the timing of surgery should be determined by considering both the severity of the condition and symptoms observed in the patient. The ACS system was introduced as a means to provide more appropriate EGSs.^9^, ^18^, ^19^ Furthermore, an increase in weekend surgeries was observed in this study after the implementation of the ACS system. This change is owing to the presence of dedicated ACS teams on weekends, which ensures prompt and efficient management of emergency cases that may otherwise face delays. Consequently, the surgical workload is evenly distributed throughout the week, enhancing overall efficiency and patient care. A meta‐analysis of 27 studies revealed that the adopting the ACS system enhanced both clinical and financial outcomes for emergency surgical patients, notably in cases of acute appendicitis, acute cholecystitis, and inguinal hernia.^20^ Based on a meta‐analysis of 48 studies, significant effects in terms of improved access to care, fewer complications, and decreased length of stay for conditions like appendicitis and biliary disease have been observed.^21^ Additionally, a single‐centre study on acute appendicitis (after the introduction of the ACS system) reported a reduction in the length of hospital stay.^22^

Our study's primary focus was the time for patients transference from the ER to the OR. This is a crucial measure of how well the ACS system works. We found that with the ACS system, patients were transferred faster, which is essential for emergency cases. The 1‐h reduction in transfer time was directly associated with the 1‐h decrease from ER admission to the time patients were admitted by the ACS team. This improvement demonstrates that the ACS system allows for faster decision‐making and execution of emergency surgeries for patients requiring EGS in the ER. This is made possible by the constant presence of ACS surgeons in the ER. This progress ultimately demonstrated a reduction in postoperative complications. In addition, operation time was reduced when dedicated ACS surgeons performed the surgery. However, these outcomes did not decrease mortality rates or reduce hospital stays. Despite adjusting for severity, there were more admissions to the ICU in the post‐ACS group, and the high APACHE II scores of these patients, indicating a poorer overall condition, may have had an influence.

The introduction of the ACS system facilitated faster surgical decisions than in the past; however, the overall time from ER admission to operation remained lengthier than in other studies.^9^ These results may have been influenced by several factors, including the availability of ORs for emergency surgery and the availability of medical staff, such as anesthesiologists and nurses. In all three hospitals where we conducted our research, no OR was kept vacant and on standby specifically for emergency surgeries. However, we believe that if such a facility was available, we could have observed even more significant reductions in the transfer time in our study. To provide appropriate care for emergency patients, financial support for emergency standby is essential, which will enable timely treatment for those in need.

With increasing specializations and diversifications within surgery in recent years, even a board‐certified general surgeon may face challenges when dealing with emergency patients outside their specific field. Unlike conditions requiring intricate surgeries, operation time in EGS plays a crucial role in patient outcomes. This field is increasingly being recognized as a specialized field.

In our study, fewer complications were observed in the post‐ACS group, indicating that excessive workload, leading to fatigue, may contribute to postoperative complications. Despite this, no correlation with surgical complications was observed according to a meta‐analysis of surgeon's fatigue.^23^ The effects of fatigue and sleep deprivation on a surgeon's precision and concentration can be likened to impaired judgement, similar to the influence of certain blood alcohol levels, potentially compromising the quality of care provided.^24^ The presence of a surgeon dedicated to handling night‐time emergencies, combined with adequate rest, benefits patients and doctors alike. Additionally, specialists who are consistently present, even during weekends and nights, can actively commit themselves to handling patients, including performing exploratory surgeries. Although in the pre‐ACS group, decisions regarding patient treatment plans were often made by residents who may have had limited clinical experience and had other duties, in the post‐ACS group, decisions were made more swiftly by specialists with extensive clinical experience, leading to a reduction in the time taken for admissions.

In our study, no significant differences were observed between the two groups in terms of hospital stay, ICU stay, re‐operation or re‐admission rate, and in‐hospital mortality. Excluding appendicitis, where the possibility of medical treatment has been recently suggested,^25^ we anticipate that conducting additional sub‐analysis on conditions where time is a more critical factor, such as trauma and ischemia, may reveal improvements in outcomes. Further research in this direction is warranted.

Through multivariate analysis, we identified that the occurrence of complications scoring 3 or higher on the Clavien–Dindo classification is associated with several risk factors. Surgery performed by an ACS specialist was reported to be a factor in lowering the incidence of severe complications. As previously known, the ACS division specializes in EGS, offering more specialized and aggressive treatment for emergency surgeries. This underlines the necessity and significance of the ACS division in managing such cases.

The strengths of this study are as follows: First, a large sample size of over 900 participants in both groups were included in the study, assuring sufficient statistical power. Additionally, we could minimize biases associated with retrospective research through propensity score matching. Second, the participation of three tertiary hospitals in the study helped mitigate the limitations of single‐centre studies. Lastly, by encompassing various surgical emergency conditions, we were able to adequately demonstrate the advantages of implementing the ACS system.

The limitation of this study is its retrospective design, which carries the potential for selection bias. We employed propensity score matching and conducted a multi‐centre study to mitigate this drawback. Additionally, among the three centres involved in the study, two (AMC and SMC) were predominantly specialized in elective surgeries, which might have influenced the selection of emergency patients. In the future, including hospitals with regional emergency centres in multi‐centre studies would be beneficial to achieve a more comprehensive understanding. Second, to evaluate the time taken from ER admission to emergency surgery, patients who underwent emergency surgery after conservative treatment during hospitalization were excluded. Moreover, the study design may not have reflected the characteristics of all emergency surgery patients. Third, in analysing the various diseases presented to the ER, diseases related to the gallbladder and appendix, which are relatively benign, accounted for approximately 30% of the cases. This could be a factor that positively affects complications and outcomes. Therefore, it seems necessary to conduct additional research focusing solely on diseases, excluding benign ones. Additionally, the timing of surgery can vary significantly depending on the condition, and delaying surgery for certain conditions may not lead to significant differences in outcomes. This variability may be more influenced by individual surgeon experience rather than standardized criteria.

## Conclusions

The implementation of the ACS system can lead to faster surgical decision‐making and the prompt execution of emergency surgery for patients, thereby reducing postoperative complications.

## Author contributions

**Gun‐Hee Yi:** Writing – original draft. **Suk‐Kyung Hong:** Investigation. **Yang‐Hee Jun:** Data curation; formal analysis. **Sungyeon Yoo:** Data curation; formal analysis. **Jung‐Min Bae:** Data curation; formal analysis. **Keesang Yoo:** Data curation; formal analysis. **Yun Tae Jung:** Investigation. **EunYoung Kim:** Investigation. **Narae Lee:** Investigation. **Min Jung Ko:** Investigation. **Hogyun Shin:** Investigation. **Hak‐Jae Lee:** Data curation; formal analysis; investigation; writing – review and editing.

## Ethics statement

The Institutional Review Board of the National Evidence‐Based Healthcare Collaborating Agency approved this study (NECA‐IRB no. NECAIRB22‐004) and waived the requirement for informed consent.

## Conflicts of interest

None declared.

## Data Availability

Data are available under request to the corresponding author.
